# Clinical and therapeutic considerations of GIST

**Published:** 2014-06-25

**Authors:** M Gheorghe, D Predescu, C Iosif, C Ardeleanu, F Băcanu, S Constantinoiu

**Affiliations:** * General and Esophageal Surgery Clinic, „Sf. Maria" Hospital, Bucharest; ** Department of Anatomopathology, „Sf. Maria" Hospital, Bucharest; *** Department of Oncology, „Sf. Maria" Hospital, Bucharest

**Keywords:** GIST, diagnostic algorithm, treatment, genetic analysis, surveillance

## Abstract

Abstract

Gastrointestinal stromal tumors (GIST) are rare tumors of the digestive tract, with an incidence of about 1.5 per 100,000/year. Clinical features may vary depending on location, size and aggressiveness. The diagnosis is confirmed by immunohistochemistry tests that identify CD 117 or DOG1 (typical receptors/markers for most GISTs) at the level of biopsy specimen.

The treatment of localized GIST is based primarily on the surgery, while for metastatic GIST the targeted therapy with tyrosine kinase inhibitors represents the current standard. The neoadjuvant and adjuvant therapy indications guided and depending on genetic analysis included in the diagnostic and treatment algorithm as well as the strategy for cases surveillance are listed in the journal. All these data obtained from the literature have been integrated in a practical experience of 19 cases of GIST, operated in the clinic in the last 10 years for which we have proposed an adapted diagnostic algorithm.

## Introduction

Gastrointestinal stromal tumors (GISTs) are rare tumors, (<1% of digestive tract tumors) with an incidence estimated at 1.5/100,000/year [**1**]. The incidence refers only to clinically manifested cases, while microscopic lesions can be detected only at a histopathological examination. GIST arise from interstitial cells described by Cajal and they are characterized by mutations of the c-KIT and PDGFRA genes which determine oncogenesis on one hand and the possibility of a precise diagnosis and targeted treatments through specific molecules, on the other hand. The average age of occurrence of the disease is 60-65 years, with wide margins. GIST are very rare in children, and represent a distinct subset of these diseases, characterized by: female predominance, KIT/PDGFRA mutations absence, multicentric gastric location and possible lymphatic metastasis [**2**]. In short, 4 major GIST categories are described: 

 1. GIST KIT mutated that occur with different locations and represent the major subgroup (85% of GIST) [**3**]. 

 2. GIST PDGFRA mutated (5-8% of GIST) that occur generally as gastric epithelioid tumors often giant, but with favorable prognosis [4,5]. 

 3. Pediatric GIST (Carney-type: Carney triad – gastric GIST, paraganglioma, pulmonary chondromas, Carney-Stratakis dyad - GIST, paraganglioma) developed in children or adults, are distincted clinical and molecular forms [6-8]. 

 4. Neurofibromatosis, type I: characterized mainly by GIST wild-type; other mutations than KIT or PDGFRA, with intestinal predominant localization and possible multiple and with favorable prognosis [**9**]. 

**Fig. 1:**
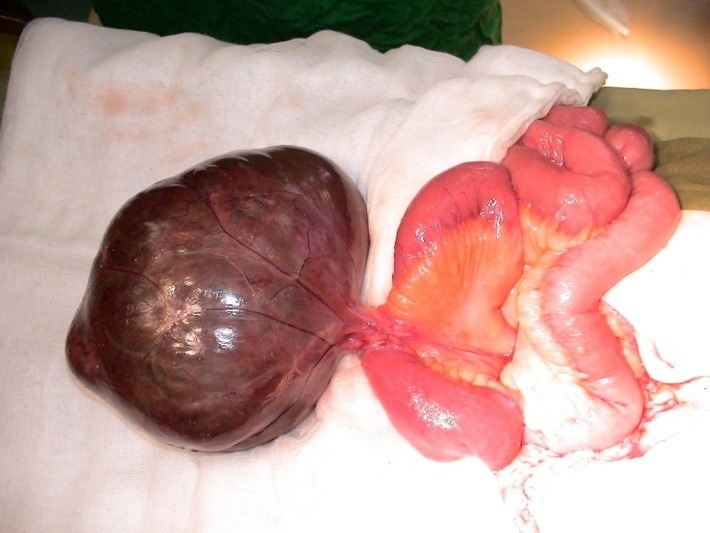
Giant intestinal GIST - intraoperative image Department of General and Esophageal Surgery "Sf. Maria" Hospital photo collection

**Fig. 2:**
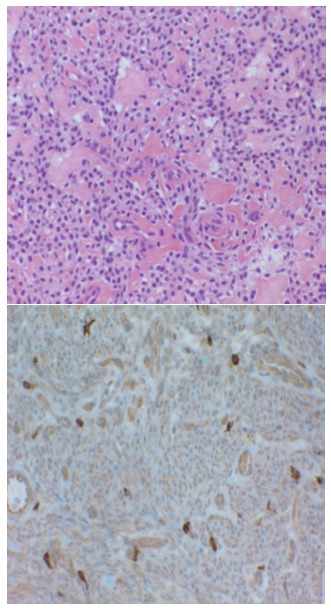
Aspects of microscopic histopathology (left-hematoxylin-eosin, 20X, right-imunohistochemistry (CD117+) Department of Anatomopathology "Sf. Maria" Hospital

 At the Esophageal and General Surgery Clinic of "Sf. Maria" Hospital in Bucharest the first case with GIST was recorded 10 years ago. Since then, the series of patients have reached 19 (12 with gastric localization, 6 with intestinal localization and 1 with pancreatic localization), a series comparable to similar centers. The disease was symptomatic: abdominal pains, anemia, fatigue, palpable tumor, upper digestive hemorrhage or intestinal obstruction. The imaging used pre and post operating consisted in esogastric barium transit, upper digestive endoscopy, abdominal echography (ECO), computed tomography (CT), positron emission tomography (PET). Confirmation was made through anatomo-pathological and immunohistochemistry examinations (**Fig. 1,2**).

 For some of the patients, the study by sequencing the oncogenes mutations and clinical-pathological correlation was managed. All patients received more or less extensive surgical resections. For some of the patients, a specific adjuvant treatment has been applied. All patients survived the surgeries. Surgery interest towards this pathology, the collaboration with the "Sf. Maria" Hospital Department of Anatomopathology Bucharest and those of Anatomopathology of "Victor Babes" National Institute of Pathology, the contribution of the oncologists trained in the treatment of this disease has allowed the centralization and comparison of data obtained as well as the communication of the results in medical journals [**10-12**]. 

 Accumulating experience of other surgical centers requires the integration of all information and matching GIST management in line with international recommendations. 

 Diagnosis of GIST 

**Fig. 3:**
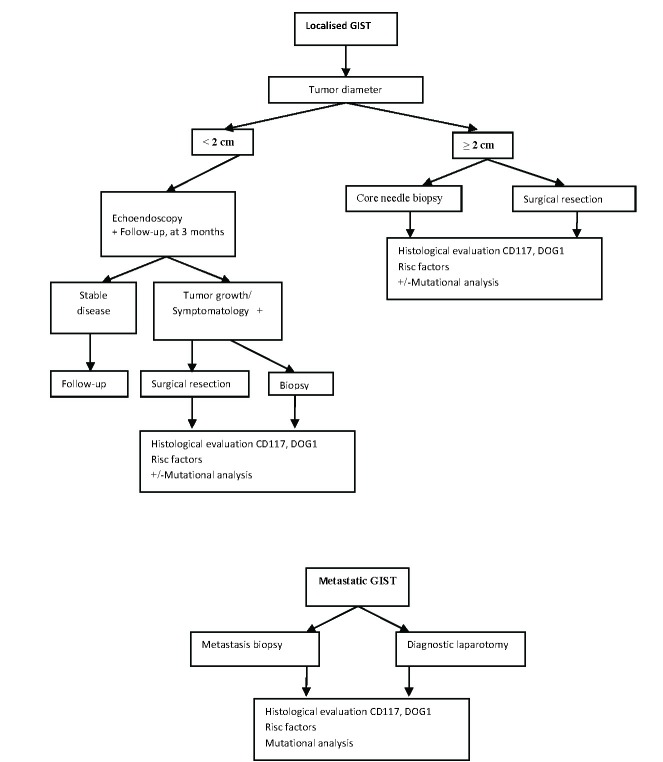
Diagnostic algorithm in GIST

 Clinical guideline on the diagnosis of GIST is suggested by the presence of a large tumor often paucisymptomatic, or with a non-specific symptomatology, with long time evolution, not being accompanied by an altered general state, specific in neoplasia. Diagnostic approach differs in GIST, being mainly linked to the size of the tumor (**Fig. 3**). 

 Thus, for nodules under 2 centimeters, the standard approach is the evaluation by eco-endoscopy and annual follow-up. For patients showing an increase in size or the appearance of symptoms related to the tumor during follow-up, the biopsy or the excision of the tumor are recommended. Alternatively, the decision may be modulated with the patient along with histological evaluation, depending on age, life expectancy, comorbidities. If the supervision way is chosen, there is not a unitary attitude with regard to follow-up. A reasonable solution should be early reevaluation at 3 months and then, if stable, a program of more relaxed follow-up could be proposed. For rectal localizations or in the recto-vaginal space, standard approach is the tumor excision/biopsy after eco-endoscopic evaluation, no matter the tumor size, due to the high risk that characterizes this localization and the important surgical implications as well. In these cases, the follow-up may be an option for smaller tumors and in some clinical contexts. 

 The standardized approach to the nodules suspected to be GIST, ≥ 2 centimeters is the tumor biopsy/excision, because if they are GIST, they have a higher risk of progression. If the structure is not accessible to endoscopic evaluation, the excision - by laparoscopy or laparotomy - in order to obtain a specimen for diagnosis, is the standard approach. In the case of large tumor masses that would require multivisceral resection, multiple biopsies, core needle biopsies, obtained under the eco-guide or percutaneous under echographic guidance or CT are recommended. They can allow the surgeon establish an optimal therapeutic plan, according to the histological diagnosis, avoiding surgery in cases such as lymphomas, mesenteric fibromatosis and germ cell tumors. If the procedure is performed correctly, peritoneal contamination risk is negligible. 

 In the case of metastatic clear determinations, the biopsy of the metastasis is usually sufficient and the patient does not require diagnostic laparotomy. The tumor fragment will be subjected to genetic analysis. 

 In terms of imaging diagnosis, especially when an experienced radiologist is involved, radiological semiology of GIST, can be highly suggestive for this kind of neoplasia: non-homogeneous tumor, the presence of a limiting capsula, the lack of the locoregional adenopathies, elements obtained by CT scan/magnetic resonance imaging (MRI) which come in addition to an abdominal ECO. The role of Fluorodeoxyglucose- PET-CT (FDG-PET-CT) in GIST diagnosis was not proved, the investigation is especially useful in assessing tumor response after the setting of targeted therapy. With regard to the preoperative staging, starting from the fact that the recurrences or the metastases of GIST concern particularly the peritoneum and the liver, the useful investigations in the characterization of the extension as well as the follow-up of the disease are mainly, the abdominopelvic CT with contrast substance, while the MRI or the ECO with contrast substance represent the alternatives. The MRI offers more accurate preoperative data for rectal GIST. Thoracic CT and routine biological tests complete preoperative lesions assessment. 

 Anatomopathological diagnosis is defining for GIST and leans on histopathological features and particularly on immunohistochemical ones: CD117 and/or DOG1 [**13-14**]. The majority of GIST are positive for CD117, but 5% of them are negative. Histological parameters such as the number of mitoses (mitoses number/50 HPF), tumor size and location of the tumor are taken into consideration at the time of diagnosis and will characterize the tumor aggressiveness and prognosis of the disease. 

 The diagnosis goes on for genetic characterization of GIST, mutational analysis confirming the diagnosis, particularly in CD117/DOG1 negative cases and having a predictive role in terms of sensitivity to specific treatment. The inclusion of molecular analysis in the diagnostic algorithm should become a standard practice with the exception of GIST with rectal localization, and GIST < 2 centimeters in diameter, for which a medical therapy is unlikely. The centralization of mutational profile results in specialized centers may be of great utility and a second opinion from an expert in sarcomas is recommended for all cases in which the diagnosis is specified outside a reference center. 

 The use of a unique way of reporting the anatomopathological result of GIST examination is necessary for the collection and reporting of data and we proposed and used a standard histological report prototype in Clinic (**Table 1**). 

**Table 1:** Histological report prototype

Tumor location Cellular type	spindle-cell	epythelioid-cell	mixed type
Number of mitosis - 50 high power fields (400x magnification)			
Capsule efraction	+/-		
Necrosis	+/-		
Vascular invasion	+/-		
Parietal invasion	+/-		
Resection margins status(invasion)	+/-		
Type of resection	R0/R1		

 Staging the GIST and classifying the risk of tumor progression 

 The TNM classification is not used in GIST. Unlike the digestive tract carcinomas which are characterized by local invasiveness (category T), as well as dissemination by lymphatic and vascular paths (category N and M), GIST do not metastasize through a lymphatic way, but only sanguine and peritoneal ways. As for soft tissues sarcomas, TNM criteria are not useful and just come to overlap the histological criteria proposed by various authors, in order to characterize the tumor aggressiveness and malignant potential of GIST [**15**]. The histological criteria accepted are: mitosis rate, tumor size and location and represent prognostic factors. Additional prognostic factors are the resection margins status and tumor rupture. Because the tumor rupture represents a negative very important prognosis factor, pre or intraoperative occurrence must be reported [**16**]. In recent years, several classifications have been proposed to stratify the risk of recurrence of localized GIST. In turn, authors like Franquemont, Fletcher - National Institutes of Health (NIH) and Miettinen - Armed Forces Institute of Pathology (AFIP) included classifications parameters such as the following in relapse risk: the tumor size, the number of mitoses, tumor proliferation index and tumor localization [**17-22**]. 

 The European Society for Medical Oncology (ESMO) recommended the use of AFIP system, recognizing its advantages (4 classes of risk with 8 subgroups); NIH system, even if it is more convenient, can lead to errors of over evaluation of malignancy grade for gastric tumors or under evaluation of non-gastric tumor [**23**]. Other authors have proposed a system of nomograms to estimate the risk of tumor progression. This system highlights the importance of tumor location for risk assessment as well as the classification of the AFIP [**24**]. 

 In an attempt to adjust the NIH System, Rutkowski has demonstrated that new parameters such as incomplete resection R1 and tumor rupture are both associated with unfavorable developments [**25-26**]. Furthermore, Takahashi and collaborators have suggested the inclusion in the risk system of a particular group: the malignant clinical group would contain patients with peritoneal disseminations; metastasis and invasion in neighboring organs or tumor rupture [**26**]. Despite the clinical invasive manifestation and extreme dissemination, these tumors may present a less aggressive histology. A more recent proposal of Joensuu, using NIH criteria, adds to the two parameters of the NIH classification, the tumor size and the mitotic activity, tumor rupture as being an independent risk factor. He also places the group of non-gastric tumors in the high risk group which reflects the influence of the AFIP system [**27**]. 

 In conclusion, the classification of AFIP (Armed Forces Institute of Pathology) is widely used and incorporates the primary tumor location, the number of mitoses and tumor size as main prognostic factors [**3-28**] (**Table 2**). 

**Table 2:**
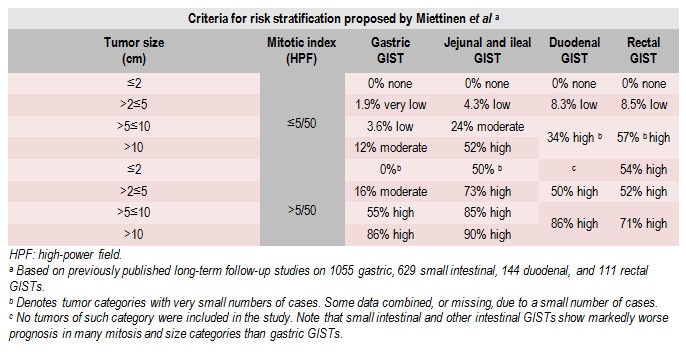
Criteria for risk stratification

 GIST’S are generally located at the intestinal level (30% of GIST) have a more aggressive behavior compared to those located at the gastric level (60% of all GIST) which have the same dimensions and number of mitoses. Using these parameters, it is important to point out that the mitotic index and tumor size do not vary linearly, such that risk stratification should be interpreted judiciously. In addition, the presence of tumor rupture exceeds from the point of view of severity the other prognostic factors and represents an independent risk factor, although it was usually described in the case of large tumors. In these cases, the patient is included in the highest degree of risk. 

 Mutational status has not yet been incorporated into the classification of the risk of recurrence, although genotypes with distinct pathological behavior have been described (**Table 3**). The application of the increasing scale of this analysis can provide future data with a statistically significant impact for their inclusion in the classification schemes of the risk of recurrence. 

**Table 3:**
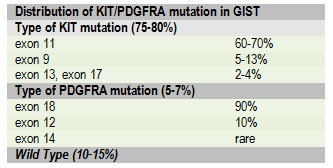
Criteria for risk stratification

 The GIST treatment 

 The participation of a multidisciplinary team in the treatment of GIST is necessary, consisting of: pathologist, radiologist, surgeon and oncologist. Just as important, is addressing this disease for sarcomas and GIST in reference centers or in networks capable of providing expertise through the approach of a large number of cases per year. 

 For Localized GIST 

 Standard treatment for located GIST is represented by the completed surgical resection, R0. If the laparoscopic resection is taken into account, it must comply with the oncologic surgery principles [**29**] (**Table 4**). 

**Table 4:**
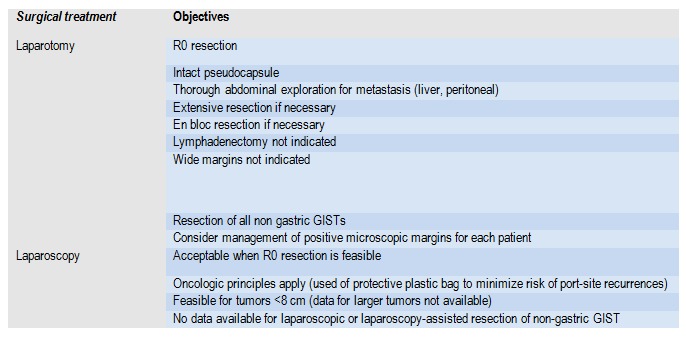
Objectives of surgical treatment in GIST

 Laparoscopic approach is not recommended in case of large tumors, due to the risk of tumor rupture associated with a very high risk of relapse. Surgical procedures are different depending on the organ involved, location and size of the tumor. Wedge resection is the most common option for gastric GIST and segmental resection is used for GIST located on the small intestine. For larger tumors, located on small gastric curvature and/or with pylorus invasion, the distal gastrectomy may be an option. Total gastrectomy may be taken into account depending on the location and GIST extension (esogastric junction). 

 The decision of an R1 resection must be discussed with the patient and accepted by him where R0 resection involves major functional sequels, or is not technically possible and preoperative treatment did not have results or cannot be administered. This attitude is especially recommended for low-risk lesions for which R1 resection is not associated with a deterioration of global survival. If a resection R1 was performed, re-excision may be an option, in terms of identifying the initial situs of the lesion and in the absence of other major functional alterations consecutive to operation. The prognostic importance is given by macroscopically full surgical resection, in order to obtain negative microscopic margins and with the avoidance of the tumor rupture. In localized GIST, the risk of relapse is substantially defined by classifications in use today. For patients at high risk of relapse, adjuvant treatment with Imatinib is the standard. Adjuvant treatment with Imatinib for a period of 3 years offers a superior overall survival in comparison with 1 year in patients with high risk of relapse [**30-31**]. Adjuvant treatment is not necessary for GIST with low risk. For those with intermediate-risk, the decision of treatment is questionable [**32**]. Mutational analysis is very important in the clinical decision concerning an adjuvant treatment. Table 5 highlights mutations in GIST and adjuvant treatment indication. 

**Table 5:**
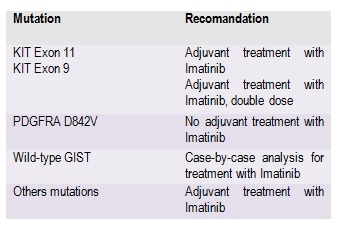
Mutations in GIST and adjuvant treatment indication

It has reached a consensus regarding patients having GIST with mutations at the PDGFRA D842V level, who will not receive adjuvant therapy, because of a lack of sensitivity of this genotype both in vitro and in vivo. Taking into account the data showing sensitivity to elevated doses of Imatinib 800 mg/day, for the cases with mutations at the KIT exon 9 level, the using of these doses as adjuvant therapy for these patients was agreed [**31-33**]. 

 Concerning the GIST wild-type which have a low sensitivity to Imatinib, due to mutations at the other genes level than KIT or PDGFRA, there is not a consensus regarding adjuvant treatment. These tumors are rare, occurring mostly in children, often asymptomatic, included in GIST syndromes and additional study is needed to conclude the therapeutic attitude. 

 Regarding the tumor rupture that occurs intraoperative, the event is considered a contamination of the peritoneal cavity, which puts these patients at a very high risk of relapse. Therefore, the treatment with Imatinib is mandatory in these cases. The optimal duration of treatment is undetermined because these patients should be considered as patients with metastasis. 

 A neo-adjuvant treatment is recommended where R0 surgery is considered possible and with a high degree of safety (less risk of bleeding or rupture of the tumor) just after tumor cytoreduction [**34-35**]. Maximum response to treatment occurs generally after 6-12 months of treatment, the surgery being performed after this period. Follow-up and assessment of the response to a neo-adjuvant treatment is done through a series of imaging examinations in one month since the beginning of the treatment, PET-CT having a proven role in the assessment of treatment response. The evaluation of the early response to a neo-adjuvant treatment is compulsory, so the surgical act should not be postponed for those patients who do not respond to therapy. In case of lack of the possibility for mutation analysis, imaging can be used for quick assessment of the disease response in a few weeks. There is no data to indicate when the treatment with Imatinib must be stopped, pre-operatory. A treatment stop is accepted with 2-3 days before the surgical act, with restarting therapy in 2-3 weeks post-surgery. 

 Metastatic GIST 

 For locally inoperable advanced cases, and metastatic GIST, Imatinib mesylate - a tyrosine kinase inhibitor (TKI) - is the standard treatment [36-40]. Before the advent of molecularly targeted therapy with TKI, efforts to treat GIST with conventional cytotoxic chemotherapy were useless. For the first time, Joensuu and collaborators have demonstrated in 2001, the effect of this TKI on tumor size reduction in the treatment of GIST [**41**]. Shortly after its introduction, initially in clinical trials, Imatinib was used systematically in the case of patients with GIST. This strategy is applicable also in patients with presented metastasis and to whom the primary lesion has been removed, although the surgery as primary therapeutic act is not recommended in these cases. 

 At the start of the treatment, the patient will be advised regarding the importance of compliance to the proposed therapy, as well as regarding the interactions with other drugs, food, and the side effects that may occur. Dose levels should be adjusted depending on the presence of side effects, the dose reduction or discontinuation is required if severe, persistent toxicity occurs. Retrospective studies demonstrate that plasma sub-optimal levels of Imatinib are associated with poor results [**42**]. Thus, the determination of the Imatinib plasmatic levels should be useful for a better adaptation of the doses in the following cases: a) patients with concomitant treatment interacting with Imatinib b) presence of toxicity-associated phenomena, c) tumor progression at doses of 400 mg/day which cause an increase of the dose to 800 mg/day. 

 For patients with sensitive GIST to Imatinib treatment (Kit exon 11 mutated), the recommended dose is 400 mg/day. In terms of patients with mutations of the exon 9 KIT, standard treatment is a double dose 800 mg/day of Imatinib, according to studies showing better survival without relapse [**43**]. The treatment should be continued for an indefinite period, its disruption leading to rapid tumor progression in almost all cases, even in cases with previous tumor excision [**44**]. 

 There are also patients with GIST resistant to Imatinib (genotypes: PDGFRA-D842V etc.) in which second-line or third-line therapies are set up in specialized centers or in clinical trials. 

 In the absence of a genetic mutation analysis of GIST performed in advance, susceptibility or resistance to treatment is assessed by tumor response to be monitored in the early stages of treatment and the follow-up is mandatory throughout the therapy because the risk of progression under treatment persists over time. In the case of a tumor response, the monitoring response can be switched from 3 to 6 months and then annually, especially after 5 years of treatment, preliminary data suggesting that the risk of relapse of disease decreases over time. 

 The importance of the surgical treatment in the metastatic GIST is in debate. Complete excision of the residual metastatic disease has been proven to be in connection with a good prognosis, proving that the patient responds to Imatinib, but it remains to be underlined whether these results are due to surgery or the selection of patients [**45-47**]. Randomized trials have not been feasible in this regard. Therefore, at present, surgical option must be individualized after discussion and consent of the patient. Surgical excision of stable disease or disease with limited progression has not been associated with a long survival more than in the case of exclusive treatment with Imatinib. The attitude must be adjusted, depending on the patient, between continuing the treatment or the limited tumor excisions. 

 In the case of tumor progression of metastatic GIST, under treatment with Imatinib, a standardized approach is doubling the dose, except in resistant cases (resistant mutations - primary resistance) [**39-40**]. In the case of progression of the disease or intolerance to Imatinib (a very rare occurrence), the standard treatment for second-line is Sunitinib [**48**]. The data available showed that oral treatment regimen, continuously dosed, with a low dose of 37.5 mg, can be effective and well tolerated, although there are no randomized comparative studies. Thus, this treatment option can be discussed as an option for custom cases [**49**]. After the failure of Sunitinib, Regorafenib is effective in prolonging survival without tumor progression, a fact proven by a prospective placebo-control randomized study [**50**]. If it is available from the commercial point of view, this therapy is recommended as the third-line treatment for patients with failure after Imatinib and Sunitinib. 

 In principle, all the patients with metastatic GIST should be included in clinical trials of new treatments or therapeutic combinations. On the other hand, the maintenance treatment with a TKI agent, even in the progression of the disease, can slow progression, as opposed to stopping treatment (in the absence of other therapeutic solutions). At the same time, the use of combinations of TKI outside of clinical trials should be discouraged due to the considerable potential toxicity. 

 Follow-up 

 There is no published data that indicate the optimal routine follow-up of patients with localized disease, surgically treated. The recurrences occur mostly in the liver and peritoneum, rarely to the bone level or in other locations. Mitosis rate indicates the speed at which the relapses will occur. Thus, the assessment of risk based on mitosis rate, tumor size and location can be useful in follow-up policy choice. High-risk patients usually have relapses after 1-2 years of adjuvant therapy. Low-risk patients usually have relapses later or not at all. Therefore, follow-up routines differ. Optimal follow-up is not known. For example, in certain clinics, high-risk patients are followed up through CT or MRI at 3-6 months for 3 years during adjuvant treatment (with a clinical surveillance for adverse effects of therapy). Then when stopping the treatment at 3 months for 2 years, then at every 6 months up to 5 years and then annually for the next 5 years. For low-risk tumors, CT or MRI exam at 6-12 months to 5 years is recommended. For GIST with very low risk, a routine follow-up is not recommended, although it is accepted that the risk of relapse is not null. What must be taken into consideration is the fact that in the context of a long-term follow-up, the radiation X poses a problem due to repeated examinations and MRI therefore represents a viable solution. 

 Generally, for patients with GIST operated or not, who have received targeted therapy, the management requires a standardized imaging follow-up algorithm for both staging and tracking, which allows a proper evaluation of the response by following up the size of the tumor, and the tumor density. RECIST criteria, appreciating the tumor size, significantly underestimate the response to Imatinib in GIST [**51**]. The best way to assess the answer by imaging CT is by evaluating the subjective criteria such as changes in the modification of tumor nodules, the density and the tumor vascularization. Criteria that use a combination of the tumor density > 15% and the modification of tumor size > 10% are promising in terms of early response evaluation and also have an excellent prognosis role. Choi and collaborators propose such adapted criteria, synthesized in Table 5 [**52**] (**Table 6**). 

**Table 6:**
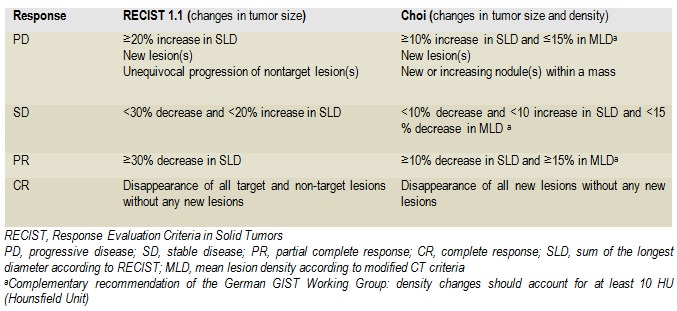
Response criteria after RECIST1.1 and Choi classifications

 Tumor sensitivity is translated as the reduction of the tumor size in most patients. For some patients, the density change of tumor may occur, observed through CT or these changes of density may precede a decrease in tumor size. In particular, even if the size of the tumor growths, a decrease in the density signifies present tumor response [51,52]. 

 The appearance of new lesions can be caused by the decrease of the radiological density visible through CT. FDG-PET-CT has proven to be very sensitive for early assessment of treatment response and can be used in unclear cases where early response assessment is most useful (preoperative cytoreduction treatment). A small proportion of GIST does not present FDG uptake. Alternatively, MRI or ECO with contrast substance can identify changes in tumor size or density, consequently the answer to the treatment. The absence of tumor progression, after months of treatment also signifies the present tumor response. 

 On the other hand, the tumor progression, therefore the treatment resistance, is not always accompanied by the growth of tumor. Tumor density growth inside the initial lesion means progression. A typical pattern is a nodule within the tumor mass "nodule within the mass"; in that nodule a part of the lesion, which has not responded to treatment, becomes hyperdense. 

## Conclusions

GIST is a rare pathology, discovered relatively recently, with a multimodal treatment that requires a multidisciplinary team. The progresses in relation to the discovery of pathogenesis of disease by identifying genetic mutations that underlie the occurrence and malignant behavior as well as the discovery of modern and effective treatments addressed to specific molecular targets provided spectacular results in terms of survival in time. Despite the fact that the series of patients are limited, multicenter studies and consensus conferences have optimized the strategies for diagnosis, treatment and follow-up for these patients.

 Far from being finished, further researches will try to integrate the mutational analysis in the diagnosis algorithms in the attempt to individualize the characteristics of each case, to ensure a personalized treatment.

 Surgical treatment remains the main procedure for localized forms. For more advanced forms, the role of surgery will be defined for each patient.

 What must be emphasized is the necessity of addressing this disease to specialize for GIST centers or networks, for full evaluation and appropriate treatment, as well as for the achievement of significant series for communicating results, for the benefit of patients.
